# Percutaneous endoscopic necrosectomy in a patient with emphysematous pancreatitis: A case report

**DOI:** 10.1097/MD.0000000000027905

**Published:** 2021-11-19

**Authors:** Shin Hee Lee, Kyu-hyun Paik, Ji Chang Kim, Won Suk Park

**Affiliations:** a Division of Gastroenterology, Department of Internal Medicine, Daejeon St. Mary's Hospital, College of Medicine, The Catholic University of Korea, 64, Daeheung-ro, Jung-gu, Daejeon, Republic of Korea; b Department of Radiology, Daejeon St. Mary's Hospital, College of Medicine, The Catholic University of Korea, 64, Daeheung-ro, Jung-gu, Daejeon, Republic of Korea.

**Keywords:** acute necrotic collection, case report, emphysematous pancreatitis, percutaneous pancreatic necrosectomy, ultra-slim endoscope

## Abstract

**Rationale:**

Emphysematous pancreatitis, a rare complication of acute necrotizing pancreatitis with a high mortality rate, is associated with gas-forming bacteria. When using the step-up approach for treating emphysematous pancreatitis, it is preferable to delay drainage interventions for 4 weeks. However, percutaneous drainage may be necessary, even in the early phase of acute pancreatitis, for a patient whose sepsis deteriorates despite optimal medical management. Percutaneous drainage can then be followed by endoscopic necrosectomy through the percutaneous tract.

**Patient concerns:**

A 52-year-old man was transferred to our hospital for treatment of sepsis and multiorgan failure associated with emphysematous pancreatitis.

**Diagnosis:**

An abdominal computed tomography scan had shown pancreatic and peripancreatic necrosis, along with extensive gas bubbles.

**Interventions:**

Despite optimal medical management, the patient's condition continued to deteriorate, and it became necessary to insert 2 percutaneous catheter drainages (PCDs), even though the patient was still in the early phase of pancreatitis. Each PCD was upsized and irrigated with sterile saline by an interventional radiologist twice a week. The infected necrosis around the tail of the pancreas was completely resolved after PCD. However, PCD through the transperitoneal route did not resolve necrosis around the pancreatic head. Following the PCDs, percutaneous pancreatic necrosectomy using an ultra-slim upper endoscope was performed, after which the patient recovered quickly and was discharged.

**Outcomes:**

Follow-up computed tomography was performed 12 weeks after the patient was discharged, and it showed complete resolution of the walled-off necrosis. The patient's condition improved without any fluid collection or complications.

**Lessons:**

PCD can be used in the early phase of emphysematous pancreatitis for patients who continue to deteriorate due to sepsis. This can easily be followed, if necessary, by percutaneous pancreatic necrosectomy using an ultra-slim endoscope.

## 1 Introduction

Emphysematous pancreatitis, a rare complication of necrotizing pancreatitis characterized by infected necrotic tissues and bubbles generated by gas-producing bacteria, has a mortality rate of 10% to 36%.^[1–3]^ In the revised Atlanta classification for acute pancreatitis, pancreatic or peripancreatic necrotic collections in patients with necrotizing pancreatitis are classified as acute necrotic collections (ANCs) for the first 4 weeks and walled-off necrosis (WON) 4 weeks after disease onset.^[4]^ In general, a sterile ANC does not warrant drainage, whereas an infected ANC may require it. In a clinically stable patient with an infected ANC, drainage should preferably be delayed for 4 weeks or more, after which the collection becomes walled off. However, early drainage in the first few weeks of necrotizing pancreatitis is required for patients whose conditions continue to deteriorate due to sepsis.^[5]^ Drainage interventions can be performed via endoscopic, surgical, or percutaneous methods. Percutaneous catheter drainage (PCD) is preferably used as a primary modality or an initial procedure in the step-up approach for treating infected ANCs.^[5]^ However, performing a percutaneous pancreatic necrosectomy to treat necrotizing pancreatitis with infected necrotic collections can be considered, especially if proper access has already been achieved by means of a percutaneous catheter drain. Herein, we describe the case of a patient with life-threatening early stage emphysematous pancreatitis that was successfully treated with PCD and subsequent percutaneous endoscopic necrosectomy using an ultra-slim upper endoscope.

## 2 Case presentation

### 2.1 Chief complaint

A 52-year-old man with a history of alcohol-induced acute pancreatitis and diabetes mellitus was transferred to our hospital for treatment of sepsis and multiorgan failure associated with emphysematous pancreatitis.

### 2.2 History of present illness

A day earlier, the patient was admitted to an outside hospital with fever and severe abdominal pain. Based on an abdominal computed tomography (CT) scan and laboratory findings, the patient was diagnosed with sepsis and multiorgan failure associated with emphysematous pancreatitis and subsequently transferred to our hospital for treatment.

### 2.3 History of past illness

The patient had a history of alcohol-induced acute pancreatitis event 2 years ago. He was also taking medications for diabetes and hypertension.

### 2.4 Personal and family history

The patient was an alcohol abuser, but not a smoker. There was no significant medical history in the family.

### 2.5 Physical examination upon admission

On arrival, the patient was receiving inotropic agents and had a body temperature of 36.5°C, heart rate of 120 beats/min, and blood pressure of 80/50 mm Hg. The patient's mental status was confused. Physical examination revealed a rigid and distended abdomen, severe tenderness with muscle guarding upon palpation, and tympanic sounds with percussion. The examination was otherwise unremarkable.

### 2.6 Laboratory examination

The results of the complete blood count test were as follows: white blood cell count, 8.1 × 103/L; red blood cell count, 3.22 × 106/L; hemoglobin, 9.1 g/dL; platelet count, 263 × 103/L and prothrombin time (INR), 1.49 (reference range 0.8–1.12). Blood chemistry testing revealed the following data: serum blood urea nitrogen, 42.0 mg/dL (reference range 6–20 mg/dL); serum creatinine, 3.46 mg/dL (reference range 0.5–1.2 mg/dL); high-sensitivity C-reactive protein, 36.12 mg/dL (reference range 0–0.3 mg/dL); serum glucose, 378 mg/dL (reference range 70–99 mg/dL); serum ketone body, 266.30 nmol/mL (reference range 0–120 nmol/mL); serum aspartate aminotransferase, 33 IU/L (reference range 8–40 IU/L); serum alanine aminotransferase, 12 IU/L (reference range 5–41 IU/L); serum amylase, 57 IU/L (reference range 41–134 IU/L); serum sodium, 125 mEq/L (reference range 136–145 mEq/L); and serum potassium, 3.9 mEq/L (reference range 3.5–5.1 mEq/L).

### 2.7 Imaging examination

**Figure 1:**
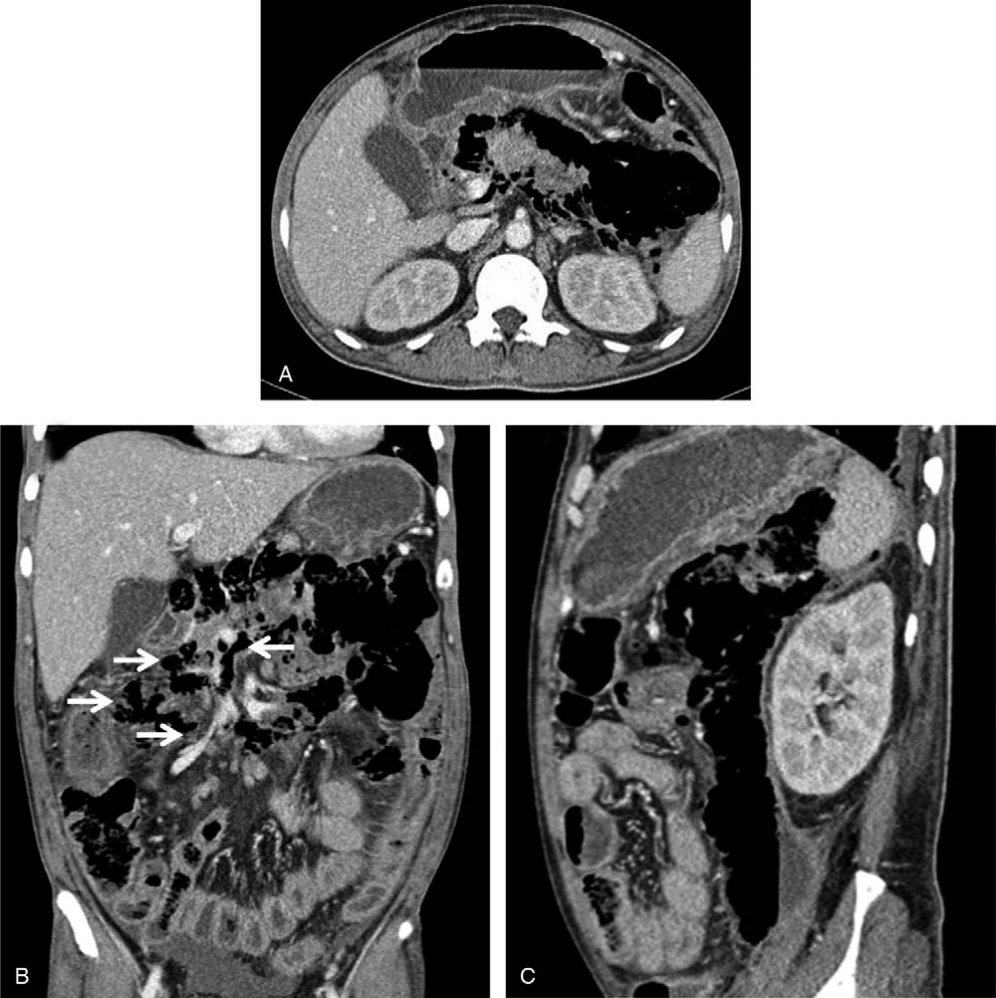
Abdominal CT showing extensive necrosis replaced with enormous amount of gas. A: An axial CT scan showing extensive pancreatic and peripancreatic gas replacing the body and tail of the necrotizing pancreas. B: A coronal CT scan showing scattered gas bubbles, along with the mesenteric root and fat (white arrows). C: A sagittal CT scan showing better visualization of the extensive gas in the left anterior pararenal space. CT = computed tomography.

An axial CT scan revealed extensive gas bubbles in and around the pancreatic necrosis (Fig. 1A) and in the mesenteric root and left anterior pararenal space. A coronal CT scan also revealed scattered gas bubbles along with the mesenteric root and fat (Fig. 1B). A sagittal CT scan showed better visualization of the extensive gas in the left anterior pararenal space (Fig. 1C).

### 2.8 Further diagnostic work-up

A Gastrografin swallow study conducted to rule out perforations did not demonstrate any leakage. Upper endoscopy also revealed no evidence of gastroduodenal lesions, such as ulcers or perforations.

### 2.9 Microbiological identification of the causative agent

Culture of the pus obtained from the catheter drainage grew *Escherichia coli*. An antibiotic susceptibility test demonstrated that the microorganism was sensitive to aztreonam, cefepime, cefotaxime, cefoxitin, ceftazidime, cefuroxime, chloramphenicol, colistin, ertapenem, and fosfomycin but resistant to ampicillin, ciprofloxacin, gentamicin, piperacillin, and tetracycline.

## 3 Final diagnosis

The final diagnosis of the patient was emphysematous pancreatitis due to *E coli*.

## 4 Treatment

**Figure 2:**
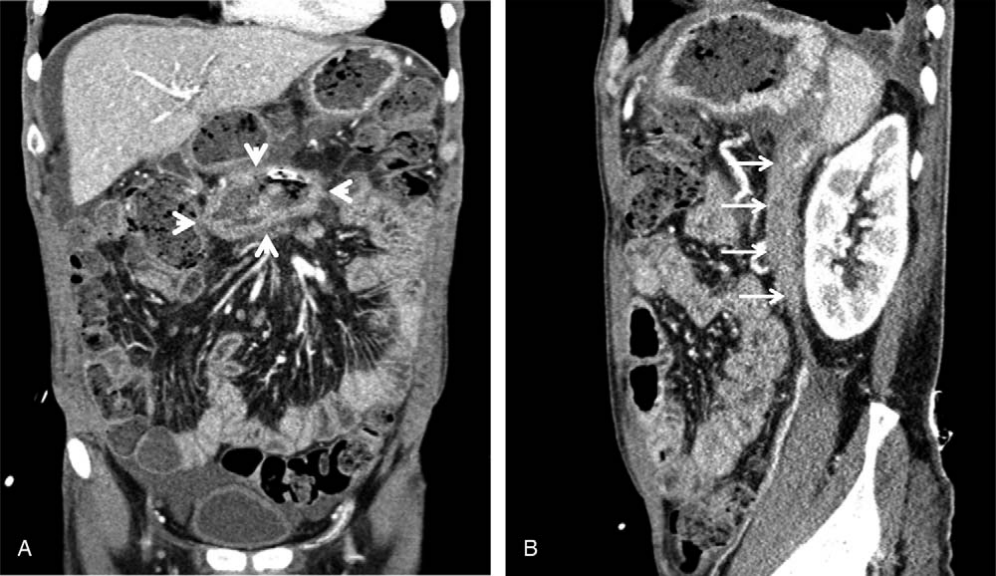
Abdominal CT showing near complete resolution of the infected WON, except around the pancreatic head. A: A coronal CT scan showing the WON with necrotic tissues and fluids still present around the head of the pancreas (white arrowhead). B: A sagittal CT scan showing near complete resolution of the gas and necrotic tissues in the left anterior pararenal space (white arrow). CT = computed tomography, WON = walled-off necrosis.

The patient was admitted to the intensive care unit of our hospital, after which we initiated vigorous fluid resuscitation and empirical antibiotic treatment with meropenem. The patient's vital signs did not improve and the fever, abdominal distension with severe pain, and confused mentality persisted for an additional 48 hours. Therefore, even though the infected pancreatic necrosis had not yet been sufficiently liquified and walled-off, we determined that it was necessary to insert a percutaneous catheter to control the infection and decompress the large amount of gas. On day 3 after admission, an interventional radiologist, guided by ultrasound, placed a percutaneous catheter into the left anterior pararenal space through a retroperitoneal approach. Three weeks later, an additional percutaneous catheter was placed around the head of the pancreas through a transperitoneal approach. The catheters were upsized to 18 Fr in the transperitoneal route and 24 Fr in the retroperitoneal route. A catheter-directed necrosectomy was performed, and the site was vigorously irrigated twice a week with sterile saline. Six weeks later, a follow-up CT revealed that most of the infected necrosis was resolved, except for the WON around the pancreatic head (Fig. 2A and B). Therefore, the retroperitoneal catheter, which showed a drainage reduction to <10 mL/day, was removed. The transperitoneal catheter remained within the medially located WON around the pancreatic head, however, catheter-directed necrosectomy was continued with vigorous irrigation performed twice a week. Nevertheless, the WON did not improve after 10 weeks, and intermittent fever persisted. Therefore, PCD alone was determined to be insufficient, and a decision to perform percutaneous endoscopic necrosectomy using the track of the previously placed catheter was made.

**Figure 3:**
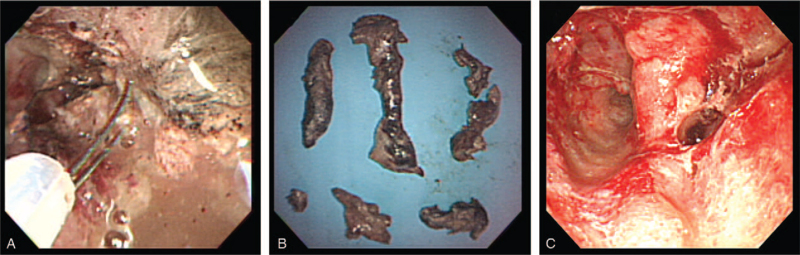
The percutaneous endoscopic necrosectomy using an ultra-slim endoscope. A: An ultra-slim endoscopic view showing purulent fluids, necrotic tissues, and clearance of the necrotic debris using a basket. B: Necrotic debris removed from the patient's pancreas. C: An endoscopic view showing viable, pink tissue with an irregular cavity after completion of the endoscopic necrosectomy.

Percutaneous endoscopic necrosectomy (Fig. 3A–C) was performed under fluoroscopic guidance with intravenous propofol sedation. The drainage catheter was then removed, and an ultra-slim upper endoscope (GIFXP 160; Olympus Co., Tokyo, Japan) with an outer diameter of 5.9 mm was introduced carefully into the necrotic cavity under fluoroscopic control. This procedure was performed with carbon dioxide insufflation. The liquid content of the WON was aspirated, and the cavity was irrigated with sterile normal saline, which was then suctioned out. The necrotic tissue was removed using various endoscopic accessories, such as biopsy forceps, a polypectomy snare, and a Memory 8-Wire Basket (Wilson Cook Medical, Winston-Salem, NC). Both necrotic materials lying free in the cavity and those attached to the wall were extracted by gently pulling on the tissues. Two sessions were performed within 3 days. Each session was terminated after all necrotic tissues were removed, and an 18 Fr catheter was reintroduced through the percutaneous tract into the cavity under fluoroscopic guidance. One week after the second session, sinus tract endoscopy was performed, which revealed near complete clearance of the necrotic tissue. Healthy granulation tissue lining the wall of the cavity was also observed, and the drainage output through the catheter was <10 mL/day. Therefore, the catheter was withdrawn, and the patient was discharged.

## 5 Outcome and follow-up

**Figure 4:**
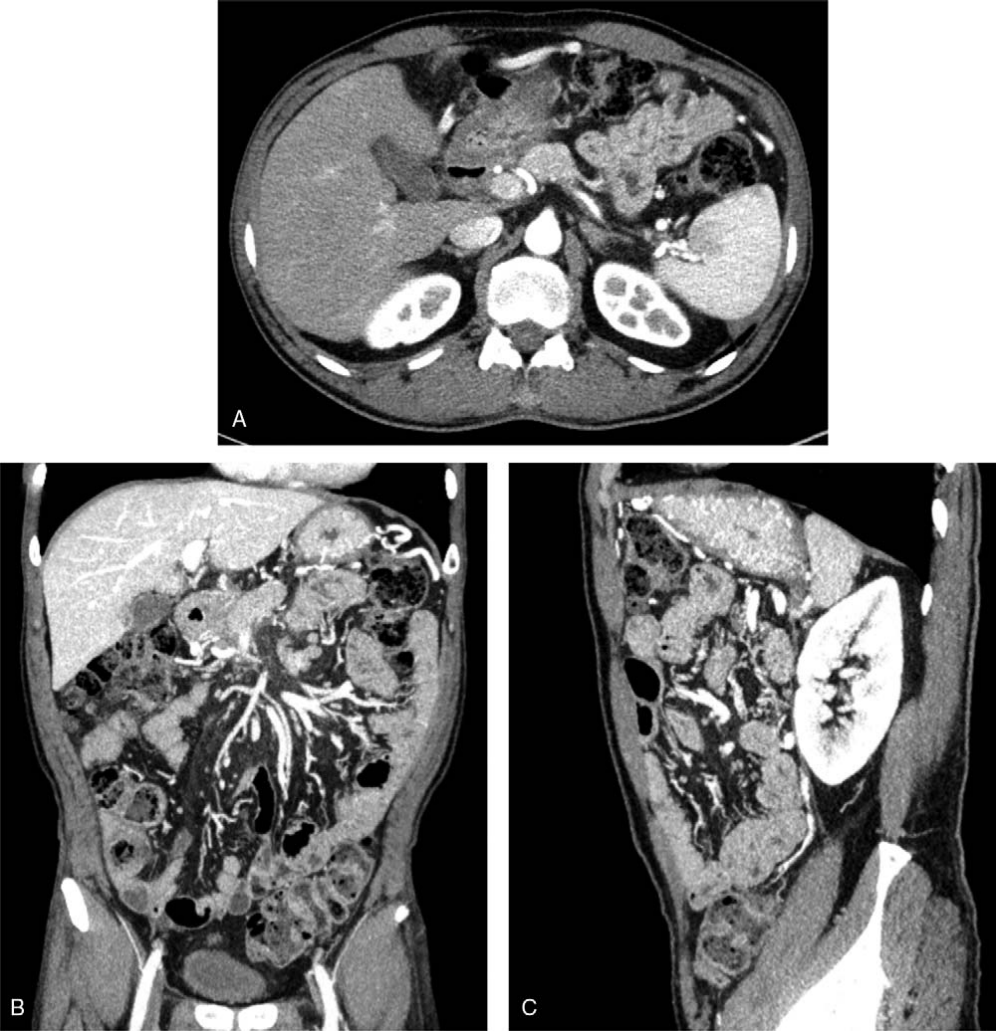
Abdominal CT showing the complete resolution of the emphysematous pancreatitis. A: An axial CT scan at the level of the pancreas body and tail. B: A coronal CT scan at the level of the pancreas head and mesenteric root. C: A sagittal CT scan at the level of the left anterior pararenal space. CT = computed tomography.

Follow-up CT was performed 12 weeks after the patient was discharged, and it showed complete resolution of the WON (Fig. 4A–C). The patient's condition improved without any fluid collection or complications.

## 6 Discussion

In the revised Atlanta classification, acute pancreatitis is subdivided into interstitial edematous and necrotizing pancreatitis.^[4]^ Infected necrotic collection is the most serious complication of necrotizing pancreatitis and is associated with a higher risk of mortality than sterile necrosis (35.2 vs 19.8%).^[6]^ Emphysematous pancreatitis, characterized by the presence of gas within the pancreatic necrotic tissue, is a serious form of infected pancreatic necrosis that has a poor outcome. The most prevalent causes of the disease are gas-producing organisms, such as *E coli, Clostridium perfringens, Klebsiella pneumoniae*, *Citrobacter* spp., *Enterococcus faecium*, *Fusobacterium*, *Pseudomonas aeruginosa*, or *Staphylococcus aureus*.^[1]^ However, there can be other causes, such as colonic or enterocutaneous fistulas, perforated duodenal ulcers, or air reflux from duodenal instrumentation.^[7]^ In the present case, upper endoscopy and Gastrografin swallow studies were performed to exclude causes other than infection by gas-forming bacteria. The prevalence of diabetes mellitus in patients with emphysematous pancreatitis is 24.1%, which is slightly higher than that of acute pancreatitis. The mortality rate is as high as 34.5%, and older age, afebrile status, and the presence of shock are associated with higher mortality.^[8]^

In the 1980s, open necrosectomies were believed to be the best treatment for necrotizing pancreatitis patients with necrotic tissue.^[5]^ However, the paradigm has shifted to a minimally invasive step-up approach, consisting of conservative treatment, percutaneous and endoscopic drainage, and minimally invasive necrosectomy.^[5]^ Recent studies have demonstrated that, compared with an open necrotomy, a minimally invasive step-up approach reduces the rates of major complications and death in patients with necrotizing pancreatitis and infected necrotic tissue.^[9–11]^

In the present case, the patient was initially managed with conservative measures, such as massive hydration and intravenous administration of inotropes and meropenem. However, his poor vital signs, fever, decreased mentality, and abdominal pain persisted for 48 hours. Therefore, it was necessary to control the infection and decompress a large amount of gas by performing a PCD, even though it was only the third day after the onset of the disease. ANCs in the early phase of emphysematous pancreatitis mostly consist of adherent solid debris. Although a recent study has reported better outcomes with lower incidences of organ failure, necrosectomies, and in-hospital mortality subsequent to early and proactive percutaneous drainage,^[12]^ it is generally believed that drainage interventions of any kind within the first few weeks after disease onset may be associated with adverse outcomes.^[5]^ Therefore, unless patients continue to deteriorate due to sepsis or abdominal compartment syndrome, it is generally recommended that drainage interventions be delayed, preferably for approximately 4 weeks after disease onset, to allow the infected acute necrotic collection to be sufficiently liquified and become walled-off.^[5]^ However, drainage may sometimes be necessary, even in the early phase of the disease, as with our patient, whose initial condition continued to worsen with the onset of sepsis.

Endoscopic transmural drainage is currently the primary modality for draining infected collections; however, for patients with infected ANCs, it is thought to induce a higher risk of cavity rupture and concomitant peritoneal contamination, and the safety data are still insufficient.^[13]^ Therefore, PCD is still preferred for draining infected ANCs, especially within the 2^nd^ or 3^rd^ week of illness. PCD was determined to be the best treatment for the patient in the current case because it was decided that endoscopic ultrasound-guided transmural drainage would be unsafe and unable to drain the area completely due to the illness being in the early phase and the magnitude of the infected necrosis, respectively.

PCD can be performed under ultrasound or CT guidance. Although the retroperitoneal approach is usually preferred, the transperitoneal approach is sometimes used. To effectively drain as much necrotic fluid as possible, the catheter can be upsized to a maximum of 28 Fr and irrigated with sterile saline several times daily.^[14]^ In a recent study, early PCD within 21 days of ANC for clinically deteriorating patients with acute necrotizing pancreatitis was shown to achieve a clinical success rate of 53.8% (42 out of 78 patients) without subsequent necrosectomies.^[13]^ With the patient in the current case, 2 percutaneous catheters were placed to accomplish drainage: one through a retroperitoneal approach to the laterally located infected necrosis and the other through a transperitoneal approach to the medially located infected necrosis. The catheters were upsized and vigorously irrigated with sterile saline 3 times a week.

The step-up approach for a patient with necrotizing pancreatitis and infected ANCs consists of PCD, followed by endoscopic necrosectomy, if necessary. Endoscopic necrosectomies are commonly performed through transgastric or transduodenal routes; however, percutaneous endoscopic necrosectomies can be performed successfully when percutaneous access has already been achieved and transmural drainage cannot be approached through the gastroduodenal wall.^[15,16]^ With the patient in the current case, the infected WON around the head of the pancreas could not be resolved with PCD and a catheter-assisted necrosectomy after 10 weeks, so a percutaneous endoscopic necrosectomy was performed. During an endoscopic necrosectomy, the forward-viewing endoscope should approach the necrotic cavity, irrigate it with saline, and clear the necrotic debris using a basket, snare, or other endoscopic accessory. If an 18 to 20 Fr percutaneous catheter has already been placed, an ultra-slim endoscope (5.9 mm diameter, 2.0 mm working channel) provides sufficient access to the necrotic cavity without any further interventions. To access the necrotic cavity with a standard endoscope (9.9 mm diameter, 2.8 mm working channel), the percutaneous tract should be upsized to 30 to 32 Fr in 1 or 2 dilation sessions or with the placement of a metal stent. Percutaneous endoscopic necrosectomies are typically performed under conscious sedation with fluoroscopic guidance, but they can also be performed at the bedside for critically ill patients with organ failure. Infected necrotic tissues can be removed using various endoscopic accessories, such as a Roth-Net retrieval device, polypectomy snare, stone retrieval baskets, or biopsy forceps.^[16]^

## 7 Conclusion

For patients with emphysematous pancreatitis, PCD to control the infection and decompress large amounts of gas is often unavoidable, even in the early phase of the illness. If percutaneous access has already been achieved, percutaneous endoscopic necrosectomy may be preferred over transmural endoscopic necrosectomy. In our experience, an ultra-slim endoscope is more suitable for percutaneous endoscopic necrosectomies than a standard endoscope because the ultra-slim endoscope has more flexibility in the necrotic cavity, does not require additional dilatation of the sinus tract, and is compatible with a variety of endoscopic accessories for patients with emphysematous pancreatitis.

## Acknowledgments

The authors thank Drs Inseok Lee (Seoul St. Mary's Hospital, The Catholic University of Korea), Dongsoo Lee, and Sang-Bum Kang (Deajeon St. Mary's Hospital, The Catholic University of Korea) for discussion and advice.

## Author contributions

Shin Hee Lee and Kyu-hyun Paik reviewed the literature drafted the manuscript; Won Suk Park performed the endoscopy, contributed to drafting the manuscript, and was responsible for revising the manuscript for important intellectual content; Ji Chang Kim performed the radiologic intervention and interpretation and contributed to drafting the manuscript. All authors approved the final version of the manuscript.

**Data curation:** Shin Hee Lee, Kyu-hyun Paik, Ji Chang Kim, Won Suk Park.

**Supervision:** Won Suk Park.

**Writing – original draft:** Shin Hee Lee, Won Suk Park.

**Writing – review & editing:** Shin Hee Lee, Kyu-hyun Paik, Ji Chang Kim, Won Suk Park.
